# Variation in Salmon Migration Phenology Bolsters Population Stability but Is Threatened by Drought

**DOI:** 10.1111/ele.70081

**Published:** 2025-02-23

**Authors:** Henry K. Baker, Mariska Obedzinski, Theodore E. Grantham, Stephanie M. Carlson

**Affiliations:** ^1^ Department of Environmental Science, Policy, and Management University of California Berkeley California USA; ^2^ California Sea Grant Santa Rosa California USA

**Keywords:** climate change, diversity‐stability relationship, freshwater fish conservation, intraspecific variation, life history diversity, movement ecology, *Oncorhynchus*, Pacific salmon, partial migration, portfolio effect

## Abstract

Intrapopulation variation in movement is common in nature but its effects on population dynamics are poorly understood. Using movement data from 3270 individually‐marked fish representing nine cohorts of coho salmon ( *Oncorhynchus kisutch* ) in California, we show that bimodal intrapopulation variation in the timing of juvenile down‐migration from their natal habitat and subsequent residence in non‐natal habitat affects growth, emigration timing, and the abundance and stability of adult returns. Non‐natal fish (early down‐migrants) exhibited more variable growth and more variable but earlier emigration to the estuary than natal fish (late down‐migrants). While natal rearing was more common, non‐natal fish were overrepresented among adult returns, and total returns were 1.4 times more stable than natal returns alone. Our results demonstrate that variation in migratory behaviour bolsters population stability. However, non‐natal rearing is reduced in low water years, suggesting that drought exacerbates population instability by reducing critical intrapopulation variation.

## Graphical abstract

We show that intrapopulation variation in juvenile movement and rearing strategies enhances population stability in endangered coho salmon near their southern range limit. However, variation in rearing strategies is nearly absent during drought years, suggesting that as drought becomes increasingly common with anthropogenic climate change, threats to imperilled fishes will be compounded by the loss of critical phenotypic variation.

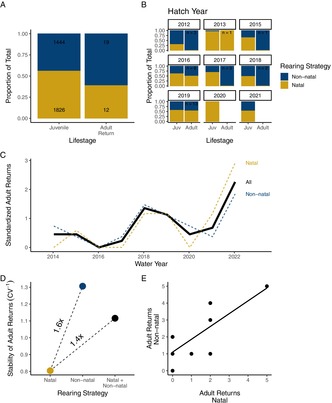

## 1 Introduction

The concept that diversity begets stability is foundational to metapopulation, community, and ecosystem ecology (Doak et al. 1998; Elton 1958; Hooper et al. 2005; Schindler et al. 2010; Tilman, Lehman, and Bristow 1998). Biodiversity‐stability relationships emerge from asynchrony in the dynamics of individual components of a system (e.g. species abundances) and the relative stability of those components (Heino et al. 1997; Thibaut and Connolly 2013). The stabilising effect of biodiversity has been termed the ‘portfolio effect’ in analogy to a well‐diversified financial portfolio, in which differential performance of distinct market sectors stabilises the portfolio yield through time (Doak et al. 1998; Tilman, Lehman, and Bristow 1998). This is a powerful concept for ecology and conservation biology, but several knowledge gaps persist regarding the occurrence, causes and consequences of diversity‐stability relationships in nature (Schindler, Armstrong, and Reed 2015).

One outstanding question pertains to the scales of biological variation that give rise to stabilising effects of diversity. Species diversity has been repeatedly shown to stabilise productivity and biomass, especially in terrestrial plant assemblages (Craven et al. 2018; García‐Palacios et al. 2018; Hooper et al. 2005; Isbell et al. 2015). There is also growing recognition that phenotypic diversity among populations promotes metapopulation stability (reviewed in Milles et al. 2023). Notable cases include Pacific salmon (*Oncorhynchus spp*.), in which stability in fishery yields has been attributed to the asynchronous dynamics of multiple, discrete spawning groups that contribute to the fishery (Brennan et al. 2019; Greene et al. 2010; Hilborn et al. 2003; Rogers and Schindler 2008; Schindler et al. 2010). However, substantial phenotypic variation often occurs within single populations, begging the question of whether intrapopulation diversity stabilises population dynamics (Bolnick et al. 2003, 2011; Milles et al. 2023).

Intrapopulation variation in movement and migration phenotypes is common in nature across diverse animal groups including insects, birds, mammals and fishes (Swingland 1983). Common manifestations of this are partial migration, in which some animals reside near their natal habitat (residents) while others undergo migrations to forage (migrants) (Chapman et al. 2011), or differential migration in which all animals migrate but the distance, stopover locations or phenology vary among subgroups (Ketterson and Nolan 1983). This latter form of intrapopulation variation has received relatively little empirical investigation, but there is reason to believe that it may be important for population dynamics. Animals that migrate at different times experience distinct environmental conditions along the migratory corridor which may impact growth potential and mortality risk, potentially decoupling population dynamics of migratory groups (Briedis and Bauer 2018).

Recent work has shown that coho salmon ( *Oncorhynchus kisutch* ) exhibit intrapopulation variation in juvenile movement timing and rearing strategies, challenging the notion that coho salmon have rigid life histories (Bennett, Wissmar, and Roni 2011; Jones et al. 2014; Koski 2009; Roni et al. 2012). In the canonical coho salmon life history, age three adults migrate from the ocean back to their natal streams to spawn in the fall and winter; fry emerge from their nests in the spring, and juveniles rear near the breeding grounds (natal habitat) until the following spring, when they migrate to the estuary as smolts and then continue out to sea (Sandercock 1991). However, variants can exhibit different strategies, including early migrations as juveniles to the estuary or other non‐natal freshwater habitats to rear for extended periods (Ebersole et al. 2006; Miller and Sadro 2003; Roni et al. 2012). Non‐natal rearing fish can also make non‐trivial contributions to adult returns, in contrast to the conventional view that they were ‘surplus’ individuals that had negligible survival (Bennett et al. 2015; Jones et al. 2014, 2021). Here, we evaluate the population effects and environmental causes of variation in movement phenology within a population. Specifically, we explore whether intrapopulation variation in movement phenology (and consequently, rearing strategies) stabilises population dynamics, and test whether environmental conditions or intraspecific competition promote this aspect of phenotypic variation.

We used detections from 3270 uniquely‐marked fish to characterise intrapopulation variation in movement timing and rearing strategies across nine cohorts of a hatchery‐supplemented population of juvenile coho salmon from the endangered Central California Coast (CCC) Evolutionarily Significant Unit (ESU) in a tributary to California's Russian River. Based on the timing of down‐migration, we classified juvenile coho salmon as natal or non‐natal fish, with the latter group down‐migrating early to spend extended residence time in the lower portion of the tributary prior to emigration to the estuary as smolts in the spring, and the former rearing in the natal habitat until smolt emigration. We characterised interannual variation in the distribution of natal versus non‐natal rearing within the population and quantified residence time spent in non‐natal habitat. We also examined the effects of rearing strategy on juvenile growth rate, emigration timing, and the abundance and stability of adult returns to the basin.

We tested the hypotheses that down‐migration timing (and, consequently, rearing strategy) would affect growth rate and emigration phenology, potentially decoupling population dynamics between groups, and that the expression of alternative rearing strategies would stabilise population dynamics. We predicted that non‐natal rearing fish would exhibit higher growth rates due to reduced competition for food resources in the colonised habitat. We also tested for environmental and ecological controls on the distribution of natal versus non‐natal rearing. We predicted that higher fall and winter flows would increase non‐natal rearing due to easier downstream passage and higher downstream habitat quality relative to lower flow years. We also predicted that higher juvenile fish densities in the natal habitat would increase the proportion of fish migrating to non‐natal rearing habitats due to higher competition in the natal habitat.

## 2 Methods

### 2.1 Study Organism and Site Description

Coho salmon occur around the Pacific Rim from Hokkaido, Japan to central California, USA. The CCC is the southernmost ESU of coho salmon and was listed as endangered in 2005 (NMFSNOAA 2005). The Russian River historically supported the largest coho salmon population within the CCC ESU, but anthropogenic disturbance associated with European colonisation severely impaired instream conditions and by the 1990's coho salmon were on the verge of extirpation from the basin (NMFS 2012).

**FIGURE 1:**
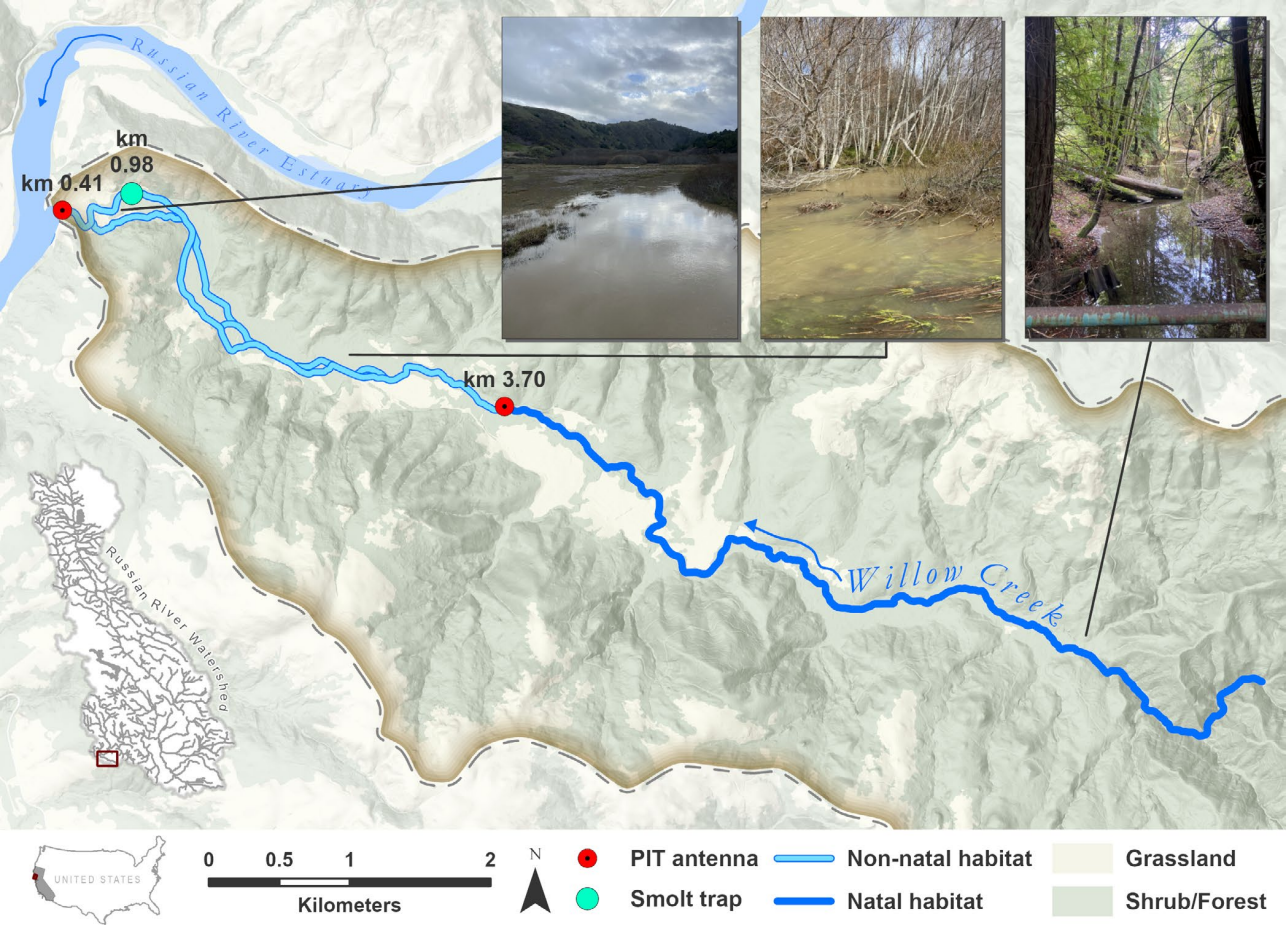
Map of Willow Creek showing the locations of PIT tag antenna arrays, the downstream smolt migrant trap, and photos showing the distinct habitat transition between the natal and non‐natal habitat within the watershed.

Our research was conducted in Willow Creek, which is the lowermost major tributary of the Russian River. It has a 22.5 km^2^ watershed and flows northwest to its confluence with the Russian River at the estuary, 4.1 km from its mouth to the Pacific Ocean (Figure 1). The creek's flashy hydrology is characteristic of Mediterranean climates (Cid et al. 2017), with high flows during the winter rainy season and low base flows with intermittent surface flow in the summer dry season. The upper portion of the creek flows through second growth redwood forests and has gravel and cobble substrate suitable for salmonid spawning. The lower portion of the creek has a braided and dynamic morphology as it flows through a flat alluvial valley with a thickly vegetated riparian zone of alder and willow, forming a network of transient channels and backwater pools that inundate the terrestrial vegetation in higher flows. The lowest portion of the creek flows through a tidal wetland that is transiently inundated during periods of high instream flow or backwater flooding from the Russian River, particularly during periods when the mouth of the estuary is closed to the ocean (Behrens et al. 2013).

The alluvial valley and tidal wetland in the lower portion of the creek–which together comprise the ‘non‐natal habitat’—are warmer, have more off‐channel habitat, and are likely more productive than the natal habitat in fall to spring rearing periods. The periodic inundation of terrestrial vegetation in the non‐natal habitat likely provides large subsidies of terrestrial insects and influx of terrestrial nutrients that increase aquatic productivity. In particular, off‐channel and floodplain habitat have been shown to yield seasonally higher growth for rearing salmon in other systems compared to main channel habitat (Brown 1985; Jeffres, Opperman, and Moyle 2008; Limm and Marchetti 2009; Swales and Levings 1989), and upper tributary sites may have lower growth potential than lower sites seasonally (Kaylor et al. 2021; see also Box 2 in Rossi et al. 2024). Other fish species in Willow Creek include steelhead ( *Oncorhynchus mykiss* ), threespine stickleback ( *Gasterosteus aculeatus* ), sculpins (*Cottus sp*.), Sacramento pikeminnow ( *Ptychocheilus grandis* ) and Sacramento sucker ( *Catostomus occidentalis* ), with greater diversity and abundance of non‐salmonid fishes in the lower portion of the creek.

Willow Creek has been the subject of ongoing restoration and salmonid recovery, including annual releases of juvenile coho salmon from a conservation hatchery using locally sourced stock [with some outcrossing to improve genetic diversity (Pregler et al. 2023)]. From 2012 to 2022, the conservation hatchery released approximately 200,000 juvenile coho salmon into the natal habitat of upper Willow Creek.

To monitor the movement of salmon within the watershed, a subset of the released fish (~15% annually) were surgically implanted with passive integrated transponder (PIT) tags at the hatchery, and a network of PIT antenna arrays was established by California Sea Grant (CSG) and Sonoma Water (SW), including antenna arrays in Willow Creek, which have been continuously operated since 2012. A dual antenna array is operated at river km 3.70 of Willow Creek, at the transition point between the breeding habitat in the upper portion of the creek, and non‐natal habitat in the braided alluvial valley and wetland reaches of lower Willow Creek (Figure 1). Another antenna array is just upstream of the confluence of Willow Creek with the Russian River estuary and detects emigration to the estuary (Figure 1). Full methodological details for monitoring by CSG and SW can be found on the CSG website (CSG 2024).

### 2.2 Data Collection

We queried SW's database on November 13, 2023 to obtain all data for fish released into Willow Creek, including detections on PIT tag antennas, and timestamped length and weight data from (1) the hatchery just prior to release into natal habitat and (2) fish captured during annual downstream smolt migration trapping by CSG and SW. In most years, a smolt trap was placed near the upper antenna site, and so it could not be used to discriminate growth patterns of fish rearing in the natal versus non‐natal habitats. However, in 2020, a trap was placed near the confluence with the Russian River estuary (km 0.98), providing 1 year of data to compare the growth of natal versus non‐natal rearing strategies.

### 2.3 Data Processing and Statistical Analysis

From 2012 to 2022, 30,101 PIT‐tagged juvenile coho salmon were released into the upper portion (natal habitat) of Willow Creek. Fish were released in either the spring (7%; 1 year) or fall (68%; 9 years) of their hatch year, or the subsequent winter/spring (25%; 5 years). We analysed data from the fall‐released fish (length = 90 ± 11 mm; weight = 8.9 ± 3.3 g) because they were the only group that had the opportunity to exhibit variation in overwintering strategies that were also released in multiple years. We filtered the dataset for fish that were detected at both the upper and lower antennas as juveniles, the former of which is necessary to classify rearing strategy and the latter of which is required to assess timing of smolt emigration to the estuary. This resulted in a final dataset that included observations from 3270 juvenile coho salmon across nine cohorts.

We characterised the timing of down‐migration based on the first detection at the upper (km 3.70) antenna array, and the timing of emigration based on the first detection at the lower antenna array. Based on the apparent bimodal pattern of the timing of down‐migration, we used finite Gaussian mixture models (fGMM) fit to the water year day (days since October 1) of down‐migration to classify individual rearing strategies as natal versus non‐natal using the R package ‘mclust’ (Scrucca et al. 2023) (see Supporting Information—Data S1 for details).

To assess the effects of natal vs. non‐natal rearing strategies on the timing of smolt emigration to the estuary, we used linear mixed models with the water year day of emigration as the response variable, rearing strategy as the fixed effect, and random intercepts by cohort (hatch year), fit using the R package ‘lme4’ (Bates et al. 2015). We compared variance between the rearing strategies using Levene's test for each hatch year using the R package ‘car’ (Fox and Weisberg 2019) and found that non‐natal fish have significantly more variable emigration timing in most years (see Results). Despite this heterogeneity of variance, we retained our linear mixed model because visual inspection of residuals showed only mild violations of distributional assumptions and linear mixed models are robust to even severe violations (Schielzeth et al. 2020). For all linear and generalized linear mixed models, we evaluated the significance of fixed effects by comparing our model to a null model fit without the fixed effect using the ‘anova’ function from the ‘stats’ package (R Core Team 2023).

To ensure that our comparisons of natal versus non‐natal fish were not biased by seasonal or site variation in detection efficiency of the PIT antennas, we compared detection efficiencies for both the upper and lower antenna arrays calculated separately for the early and late down‐migration periods (Oct–Feb and Mar–Jun, respectively) for each cohort in our dataset. Antenna efficiencies were estimated using an application of a multistate emigration model (described in Horton, Letcher, and Kendall 2011) using Program MARK (White and Burnham 1999). We then evaluated the fixed effects of season and site, as well as their interaction in a linear model, which showed no significant effects (see Supporting Information—Data S1).

To test whether our data on hatchery fish are representative of the behaviour of wild fish, we evaluated the down‐migration timing of a small sample (*n* = 79) of wild fish tagged in upper Willow Creek in 2020 and 2023 using the same methods as for the hatchery fish (see Supporting Information—Data S1).

We used chi‐squared tests to evaluate whether natal or non‐natal rearing was more common among juveniles. We then used a chi‐squared test to evaluate whether natal or non‐natal fish were overrepresented among adult returns relative to their distribution as juveniles. To compare growth between rearing strategies for the one cohort for which we had growth data from both groups, we used a Kruskall‐Wallis test because Levene's test showed greater variance in the non‐natal than natal fish.

To compare the stability of the diversified (natal + non‐natal) and baseline (natal only) conditions, we calculated the coefficient of variation (CV = standard deviation divided by the mean) and its inverse (stability = CV^−1^) of total annual adult returns for each group and the population as a whole (Schindler et al. 2010). For plotting annual variation in adult returns for each of the groups (natal, non‐natal and total), we standardized annual returns to the group's mean (across all return years), such that a value of one indicates that year's returns were equal to the group's long‐term mean (see Figure 5c) (Schindler, Armstrong, and Reed 2015). We used Pearson's correlation coefficient (*r*) between annual adult returns (2014–2022) from natal and non‐natal rearing groups to evaluate their (a)synchrony.

To model the effects of streamflow and population density on the proportion of fish exhibiting non‐natal rearing, we used beta regression models fit with the ‘betareg’ package in R (Cribari‐Neto and Zeileis 2010). We extracted streamflow data from the Austin Creek USGS gage using the ‘dataRetrieval’ package in R (De Cicco et al. 2024). Austin Creek is the nearest tributary on the Russian River with reliable streamflow data and serves as a reasonable proxy for Willow Creek because of its proximity and because the hydrograph of both streams are driven by precipitation in the wet season. Because non‐natal fish down‐migrate soon after being released into the natal habitat, we calculated mean daily flow for the 14‐day period following the release date (which was also representative of longer‐term conditions; see Figure S4) as our predictor variable, which we log‐transformed to improve model fit.

We also tested the effects of the abundance of juvenile fish in the natal habitat (including both wild and hatchery fish; a proxy for the strength of intraspecific competition) on the proportion of fish exhibiting non‐natal rearing using beta regression (see Supplement for calculation of juvenile abundance).

Prior to model fitting, we added an arbitrarily small number (10^−5^) to the response value for the 1 year in which the proportion of fish exhibiting non‐natal rearing was exactly zero to comply with the requirement of beta regression that response values fall in the open interval (0,1). We first tested a model with both predictors as additive fixed effects, then dropped non‐significant predictors and refit the model. We verified that dropping predictors did not reduce the information content of the model using AIC. We report results from the additive model when discussing dropped predictors, and results from the final model when discussing retained predictors. We also compared the size at release of non‐natal and natal rearing fish using a linear mixed model with hatch year as a random effect.

## 3 Results

### 3.1 Prevalence and Interannual Variation in Non‐Natal Rearing

**FIGURE 2:**
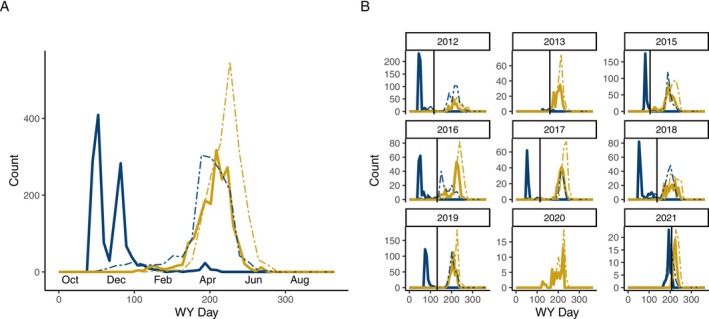
The distribution of the timing of down‐migration from the natal habitat into lower Willow Creek (solid lines) and the timing of emigration from lower Willow Creek to the Russian River estuary (dashed lines) for fish classified as non‐natal (blue) or natal (yellow) fish (A) aggregated across all years and (B) separately by cohort (hatch year). Vertical lines in (B) show the threshold for classifying rearing as natal or non‐natal.

fGMMs supported a bimodal distribution in the timing of down‐migration from the natal habitat in all years except 2020 (Figure 2). Classification error based on the identified thresholds was uniformly low (0%–5.1%) in all years where a bimodal model was supported, reflecting the distinctiveness of the constituent distributions (Figure 2). Non‐natal rearing was less common than natal rearing, comprising 44% of the sampled population across all years (*X* ^2^ = 44.6; *p* < 0.001; annual range: 0%–70%; Figure 5a).

### 3.2 Phenology of Down‐Migration and Emigration

Across the eight cohorts which exhibited both rearing strategies (all cohorts except 2020), non‐natal fish down‐migrated an average of 117 days earlier than natal fish (Figure 2). The timing of down‐migration was more variable for non‐natal than natal fish in six of the 8 years in which both strategies were present (*p* < 0.01), and the mean date of down‐migration was more variable inter‐annually for non‐natal than natal fish, though this effect was marginally insignificant (*p* = 0.08).

**FIGURE 3:**
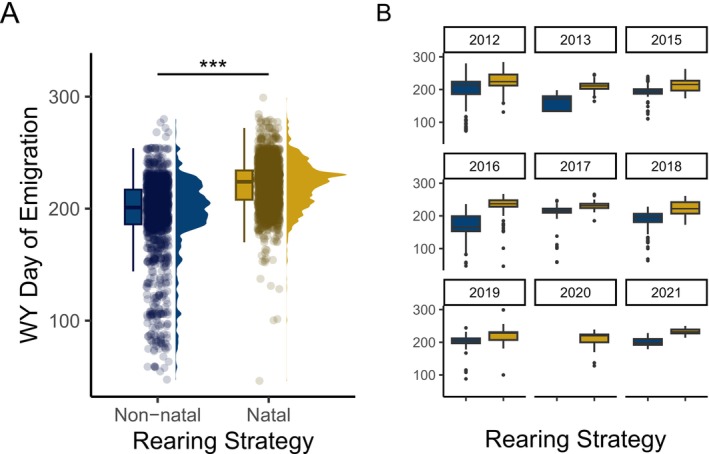
Emigration timing of non‐natal (blue) and natal (yellow) rearing coho salmon (A) aggregated across all years, and (B) separately by cohort (hatch year). Asterisks denote a significant difference with a *p* < 0.0001 from a linear mixed effects model.

Non‐natal fish spent 125 days (mean) in lower Willow Creek, while natal fish passed through relatively quickly (mean = 19 days) (Figure S2). The timing of emigration to the estuary was more variable for non‐natal rearing fish (*p* < 0.001), but non‐natal fish emigrated significantly earlier than natal rearing fish (mean difference = 34 days; LME: *X* ^2^ = 751.8; *p* < 0.001), and the direction of this effect was consistent across all years (Figure 3).

Analysis of a sample of 79 wild fish tagged in upper Willow Creek showed that wild fish exhibit similar bimodal variation in down‐migration timing as hatchery fish (Figure S5).

### 3.3 Effects of Rearing Strategy on Growth

**FIGURE 4:**
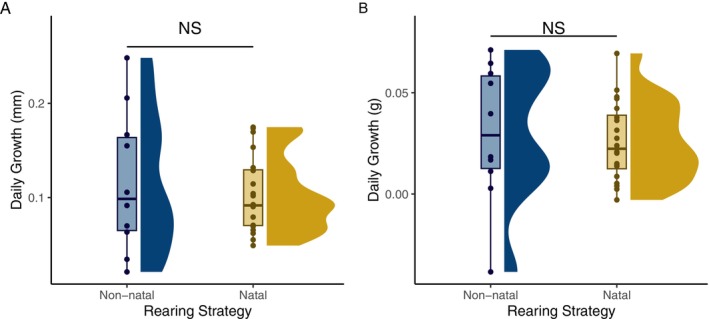
Growth in (A) fork length (mm) and (B) weight (g) for non‐natal (blue) and natal (yellow) rearing juvenile coho salmon captured at the downstream migrant trap in 2020 (hatch year 2019). ‘NS’ denotes no significant difference in central tendency from Kruskal‐Wallis rank sum test. Variance was greater for non‐natal rearing in both A and B (Levene's test for homogeneity of variance; *p* < 0.05).

Mean growth rate did not vary with rearing strategy (length: *X* ^2^ = 0.04, *p* = 0.83; weight: *X* ^2^ = 0.44, *p* = 0.51), but non‐natal rearing coho exhibited greater variation in growth rates than natal fish (length: *F*_1,28_ = 4.96, *p* < 0.05; weight: *F*_1,28_ = 5.35, *p* < 0.05; Figure 4).

### 3.4 Effects of Rearing Strategy on Adult Returns

**FIGURE 5:**
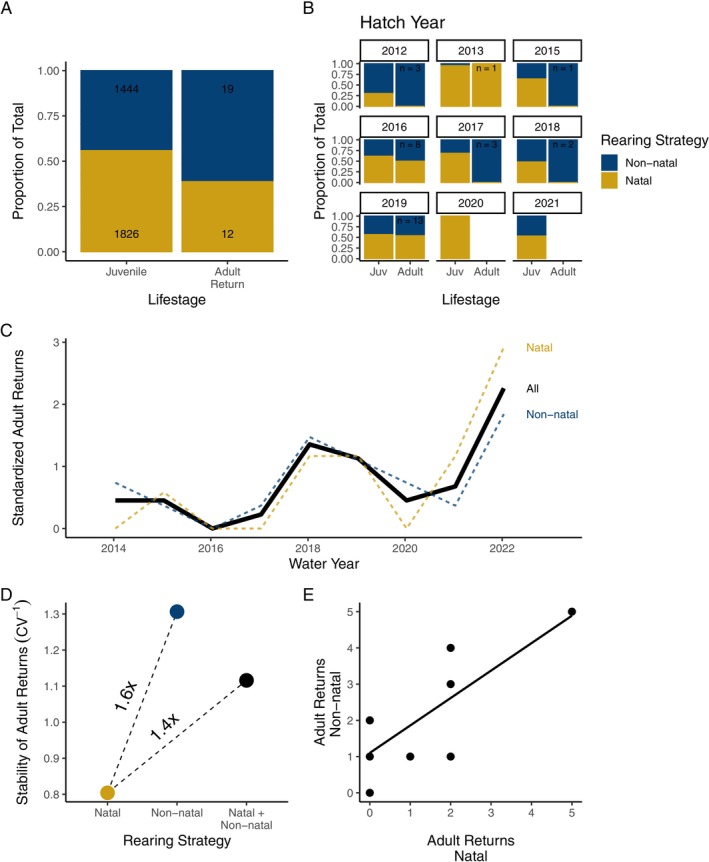
Effects of variation in rearing strategy on the abundance and stability of adult returns: (A) The rearing strategy composition of PIT‐tagged juveniles and adult returns aggregated across years and (B) separately by cohort (hatch year). (C) Inter‐annual variation in adult returns by juvenile rearing strategy, standardised to the group long‐term mean [by water year (Oct–Sep) rather than cohort because adults spend either 1 or 2 years at sea]. (D) Comparison of the stability of adult returns (the inverse of the coefficient of variation) by juvenile rearing strategy. (E) The temporal correlation of adult returns by natal and non‐natal rearing fish. Adult data represent individually marked fish with rearing strategies assigned as juveniles that survived to adulthood (see methods).

Of the fall‐released juvenile coho salmon in our dataset, 31 were detected returning to the Russian River tributaries as adults as age‐2 or age‐3 fish. Non‐natal fish were over‐represented among adult returns relative to their distribution as juveniles, comprising 61.3% of total returns (Figure 5a,b), though this effect was marginally insignificant (*X* ^2^ = 3.69, *p* = 0.055) likely reflecting the small sample size of this endangered population.

Annual adult returns to the watershed were 1.4 times more stable in the diversified condition (natal + non‐natal) than in the baseline condition (natal only), due primarily to the relative stability of non‐natal fish (Figure 5d). The dynamics of natal and non‐natal returns were only mildly asynchronous (*r* = 0.78, *p* < 0.05; Figure 5e).

### 3.5 Ecological and Environmental Predictors of Variation in Rearing Strategies

**FIGURE 6:**
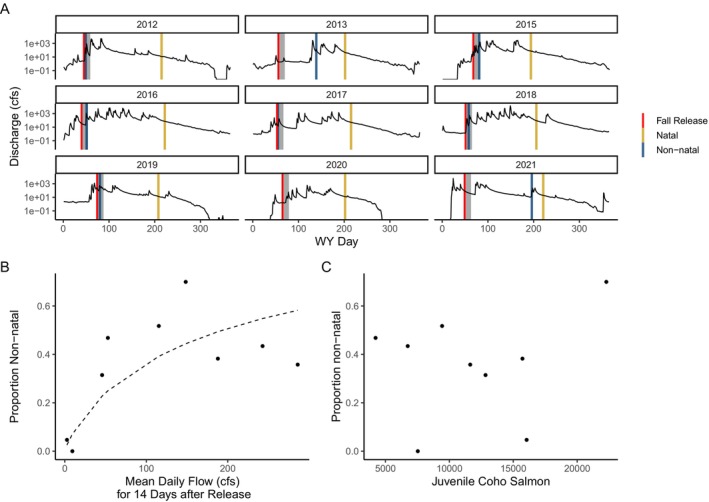
Environmental effects on the distribution of natal versus non‐natal rearing. (A) Discharge (cfs) at the Austin Creek gage (our proxy for Willow Creek) by cohort (hatch year). Red lines denote the timing of hatchery releases into Willow Creek, gray boxes encompass the 2 weeks following release. Yellow and blue lines denote the median down‐migration timing for natal and non‐natal rearing fish, respectively. (B) The effects of flow (mean daily flow for the 2 weeks following release) on the proportion of fish exhibiting non‐natal rearing. The dashed line shows predictions from the beta regression model. (C) The relationship between the number of juvenile coho salmon (including wild and hatchery fish; a proxy for competition) and the proportion of fish exhibiting non‐natal rearing.

Mean daily flow (cfs) in the 14‐day window following the release of fish into Willow Creek was positively related to the proportion of fish exhibiting non‐natal rearing (beta regression: *z* = 3.69; *p* < 0.001; Figure 6a,b). In the 2 years for which mean daily flow in this window was less than 10 cfs [water years 2014 and 2021 (cohorts 2013 and 2020, respectively), both drought years], non‐natal rearing was absent or nearly absent (< 5%) from the population [low flow in the 14‐day window was also reflective of conditions at broader time windows (2–4 months; Figure S4)]. We found no evidence that the density of juvenile salmon– a proxy for intraspecific competition– affects the proportion of fish exhibiting non‐natal rearing (beta regression; *z* = 1.30, *p* = 0.19; Figure 6c). We also found no evidence that non‐natal rearing fish were smaller at the time of release than natal rearing fish. In fact, non‐natal rearing fish were larger, though the effect size was very small (LME: *X* ^2^ = 63.4, est. = −2.6, *p* < 0.001; Figure S3).

## 4 Discussion

Previous studies have demonstrated that species diversity and population diversity increase the stability of ecosystems and metapopulations, respectively (reviewed in Schindler, Armstrong, and Reed 2015). Our results demonstrated that finer scales of biological variation—specifically, intrapopulation variation in movement phenology—bolster population stability. We showed that bimodal variation in the timing of down‐migration and the resulting residence time in non‐natal habitat affected growth and the timing of emigration of coho salmon to the estuary—two critical elements of juvenile survival. Adult returns were disproportionately comprised of non‐natal rearing fish and annual returns by non‐natal fish were 1.6 times more stable than natal fish. Finally, we showed that the expression of the non‐natal rearing strategy depended strongly and positively on streamflow, suggesting that drought may exacerbate population instability by reducing intrapopulation variation in movement phenology.

Intrapopulation variation in migration strategies is common across a wide variety of taxa including ungulates (Ball, Nordengren, and Wallin 2001; White et al. 2007, 2010), birds (Both et al. 2010; Falchi et al. 2023; Newton 2012; Pulido, Berthold, and van Noordwijk 1996; Pulido and Widmer 2005), insects (Slager and Malcolm 2015; Yang et al. 2016) and fishes (Quinn and Brodeur 1991). Our results demonstrate that this variation can have important consequences for population dynamics and should be considered in management contexts. We showed that variation in migration strategies is under strong environmental control, indicating that the negative impacts of anthropogenic environmental change on population persistence may be compounded by losses of critical phenotypic variation. On the other hand, the restoration of environmental conditions that promote intrapopulation variation may bolster population stability and accelerate species recovery (Watters, Lema, and Nevitt 2003).

### 4.1 Early Down‐Migration and Non‐Natal Rearing Is Adaptive

Our finding that non‐natal rearing juvenile coho salmon were overrepresented among adult returns may be underlain by differences in growth and emigration timing between the two strategies. Non‐natal fish exhibited a greater range of growth rates than natal fish, consistent with a high‐risk, high‐reward model in which some non‐natal fish are able to capitalize on high growth opportunities in non‐natal habitat, while others may get stranded in poor quality habitat or have high energy expenditures searching for suitable habitat. Natal fish, in contrast, may have lower growth potential but also more stable and predictable conditions without the energetic cost and risks associated with early dispersal. That said, our growth data are limited to one cohort and may not be fully representative of patterns across broader timescales.

The earlier but more variable emigration timing of the non‐natal fish may increase their probability of exiting the river during the favourable oceanic growth conditions during the onset of upwelling (Beamish and Mahnken 2001; Duffy and Beauchamp 2011; Kastl et al. 2022; Satterthwaite et al. 2014; Scheuerell, Zabel, and Sandford 2009; Spence and Hall 2010) relative to natal fish, especially in bar‐built estuaries like the Russian River in which the mouth is only transiently open for fish passage (Behrens et al. 2013).

That non‐natal fish appear to have higher performance than natal fish raises the question of why natal rearing is the dominant strategy. We might expect directional selection for non‐natal rearing, yet we see no evidence across nine cohorts that non‐natal rearing is increasingly common. In our system, gene flow through hatchery operations would limit directional selection at the subwatershed scale if non‐natal rearing is adaptive in only some of the locations. Yet, the fact that (to our knowledge) no coho salmon populations across their broad range exhibit a wholly non‐natal rearing strategy suggests that hatchery operations do not fully explain this phenomenon. Adaptive evolutionary mechanisms such as bet‐hedging (which is consistent with the higher variance in performance of non‐natal fish that we observed) or frequency dependent selection may be responsible for the maintenance of variation that we observe (Ayala and Campbell 1974; Phillipi and Seger 1989). Identifying the evolutionary drivers of intrapopulation variation should be a priority for future research.

### 4.2 Intrapopulation Diversity Bolsters the Stability of Adult Returns

Our results showed that within‐population trait variation promotes population growth and stability. While life history diversity has long been recognized as a factor promoting stability in a variety of organisms including Pacific salmon (Carlson and Satterthwaite 2011; Griffiths et al. 2014; Hilborn et al. 2003; Moore et al. 2010; Satterthwaite and Carlson 2015; Schindler et al. 2010; Thorson et al. 2014), previous demonstrations of this phenomenon have largely emphasized diversity among populations and its stabilizing effects in a metapopulation context. Distinguishing scales of variation and their effects on stability is important because eco‐evolutionary processes such as local adaptation or gene flow may differentially impact (and be impacted by) intra‐versus inter‐population diversity. Additionally, effects of stability may propagate across scales.

In other systems, additional life history variation has been observed in coho salmon, including fish that spend a second winter in freshwater before migrating to sea, fry migrants and estuarine‐rearing juveniles (Bennett, Wissmar, and Roni 2011; Gorman 2016; Jones et al. 2014, 2021; Koski 2009; Roni et al. 2012; Sandercock 1991). As each of these strategies differentially interacts with spatio‐temporal variation in resource fluxes across the riverscape (foodscapes *sensu* Rossi et al. 2024), we expect that these strategies would exhibit different growth trajectories and phenologies, decoupling their dynamics and conferring stability to the collective population.

### 4.3 Streamflow, Not Competition, Causes Variation in Rearing Strategies

Prior research has suggested that early down‐migration followed by non‐natal rearing is a consequence of competition in the natal habitat (Chapman 1962, 1966). Under this model, subordinate individuals that fail to establish territory in the natal habitat either search for new habitat downstream or are relegated to inferior portions of the natal habitat and subsequently displaced downstream during high flows (Chapman 1962, 1966; Hartman, Andersen, and Scrivener 1982; Tschaplinski 1987). Our data provided a unique opportunity to test this hypothesis, as the number of released fish varied more than sevenfold over the nine cohorts we studied, providing a strong gradient of intraspecific competition. We found no relationship between the number of juveniles present in the natal habitat and the proportion of fish exhibiting early down‐migration and non‐natal rearing, consistent with the notion that non‐natal rearing is volitional and not a consequence of competitive displacement. This interpretation is further supported by the fact that natal rearing fish were not larger at the time of release than non‐natal rearing fish, as size is a strong determinant of competitive ability and social status in coho salmon (Holtby and Healey 1990). Some of the prior work on this topic focused on smaller fish (emergent fry) and it may be that the drivers of early down‐migration vary with ontogeny.

Our results showed that streamflow, not competition, strongly controls variation in rearing strategies in the Willow Creek coho salmon population. In very low flow years, non‐natal rearing was absent or nearly absent. This may be due to passage barriers at low flow, the absence of cues to initiate downstream movement or low predictability of downstream conditions, leading to the decision to remain in the natal habitat. Future work is needed to distinguish the mechanisms driving the relationship between streamflow and non‐natal rearing.

### 4.4 Management Actions That Enhance Life History Diversity Promote Population Recovery

Our results demonstrate that phenotypic variation within a single population bolsters population stability. As such, we recommend that future research characterize important axes of phenotypic variation within populations of concern and the environmental and ecological factors that affect the dynamics of population subgroups to improve ecological forecasting and conservation decision making.

For coho salmon near their southern range limit, we showed that variation in migration phenology improves population stability but is threatened by drought. As drought becomes increasingly common with global climate change (Dai 2013), reduced phenotypic variation may compound other threats to imperilled fishes, including the loss of connectivity and rising water temperatures (reviewed in Lennox et al. 2019). On the other hand, the fact that early down‐migration is plastically expressed by a subset of the population depending on environmental conditions (sufficient streamflow) suggests that this life history strategy can be recovered with the restoration of suitable conditions. This suggests that single‐site restoration actions that promote intrapopulation diversity can promote recovery even if among‐population diversity is degraded (see also Sturrock et al. 2020).

We advocate for a phenotype management approach to restoration, in which promoting phenotypic diversity in the target species is an explicit goal of the restoration process (Watters, Lema, and Nevitt 2003). Changes to habitat conditions through restoration can alter the selective landscape, with the potential to promote or suppress phenotypic diversity. For example, restoration that expands potential recipient habitats for non‐natal rearing fish may promote alternate rearing strategies, even if those strategies are not currently observed in the impaired system. However, predicting the outcomes of restoration on phenotypic expression is not a simple task and even thoughtfully designed restoration actions may have unintended consequences. For example, changes to natal habitat intended to improve holding conditions for juvenile fish could, in theory, suppress the expression of non‐natal rearing behavior. Because of this complexity, we emphasise the need for an adaptive approach to phenotype management which requires both pre‐ and post‐project monitoring of phenotypic variation to improve restoration outcomes and population recovery.

Intrapopulation variation in migratory traits is not restricted to salmon, and may be an important determinant of population stability for a range of animals. Preserving and restoring migratory variation can improve population outcomes and can be achieved with an improved understanding of its underlying drivers (Barker et al. 2022).

## Author Contributions

All authors conceptualized the study. H.K.B. analyzed the data and wrote the manuscript. All authors reviewed and revised the manuscript. M.O., T.E.G. and S.M.C. secured funding for the project.

### Peer Review

The peer review history for this article is available at https://www.webofscience.com/api/gateway/wos/peer‐review/10.1111/ele.70081.

## Supporting information


**Data S1.** Supporting Information.

## Data Availability

Data are available at the following Dryad repository: https://doi.org/10.5061/dryad.7sqv9s521. Code is available at the following Zenodo repository: https://doi.org/10.5281/zenodo.13324366.
