# Supplementary material for: Variation in Salmon Migration Phenology Bolsters Population Stability but Is Threatened by Drought
Source: Ecol Lett. 2025 Feb 23;28(2):e70081. doi: 10.1111/ele.70081 (PMC11848020; doi:10.1111/ele.70081)
Supplement: Supplementary file 1 — Data S1. Supporting Information. [file ELE-28-0-s001.pdf]

SUPPLEMENT FOR: Baker et al. (2025). Variation in salmon migration phenology bolsters population stability but is threatened by drought. *Ecology Letters.*

*Classifying rearing strategies with finite Gaussian mixture models*

Finite Gaussian mixture models (fGMMs) model multimodal distributions as a weighted combination of Gaussian distributions (Scrucca et al. 2023), and are used in fisheries research to classify discrete traits from multimodal data, including classifying fish age from length (Sethi et al. 2017). This is done by fitting the fGMM to empirical data, simulating data using the parameters of the fitted model, identifying the threshold value that minimizes classification error in the simulated data with known group classifications, then applying that threshold to the empirical data (Sethi et al. 2017) (Figure S1). We applied this technique to our down-migration data separately for each year, using the water year day of down-migration as our modeled variable, constraining the model to two component Gaussian distributions. We also allowed for a unimodal model if that provided a better fit in a given year. For each year, we compared the uni- and bimodal models using Bayesian Information Criterion (BIC) and the classification error in the simulated data. We used a unimodal model for a given cohort if the BIC value indicated a better fit, or if classification error of the bimodal model exceeded 20%, indicative of low differentiation between the constituent distributions. When the bimodal model was supported, we classified individuals that down-migrated to non-natal habitat before and after the threshold as non-natal and natal fishrearers, respectively.

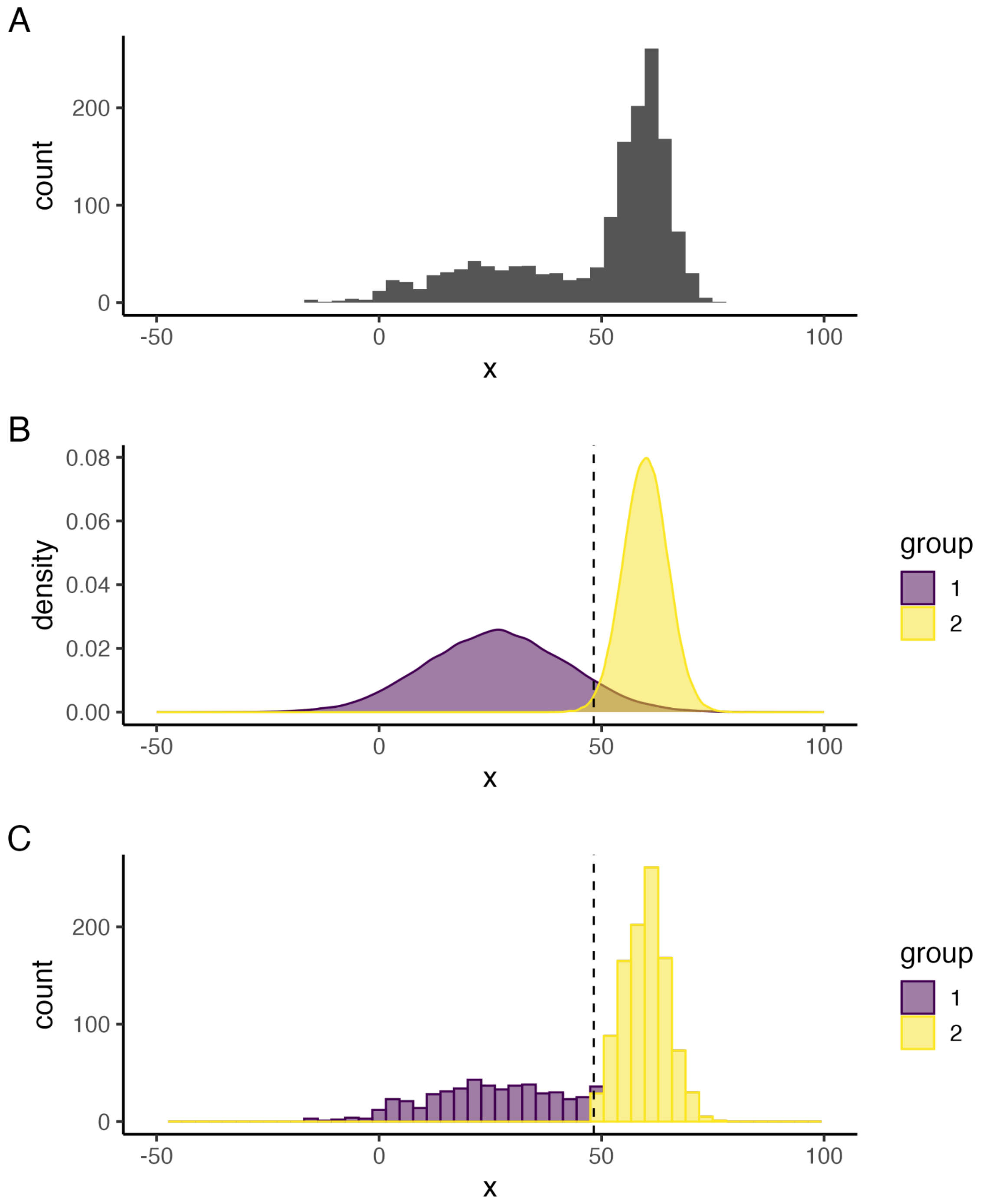


Figure S1: Example of the classification process using finite Gaussian mixture models (fGMM) fit to multimodal data. (A) An example of bimodally distributed empirical data. (B) Densities of data simulated using parameter estimates from a two-component fGMM, and the threshold value (vertical dashed line) that minimizes group classification error in the simulated data with known group identities. (C) The application of the identified threshold to make group assignments in the empirical dataset.

*Detection efficiency of PIT antennas*

Detection efficiency on the PIT antennas was generally high across both the upper and lower antenna arrays (median efficiency = 97.2%) and did not vary by season (Oct-Feb versus Mar-Jun; $p = 0.72$) or by site (upper versus lower antenna; $p = 0.30$), and there was not a significant interaction between season and site ($p = 0.72$), indicating that our comparisons of rearing groups were not biased by detection efficiency.

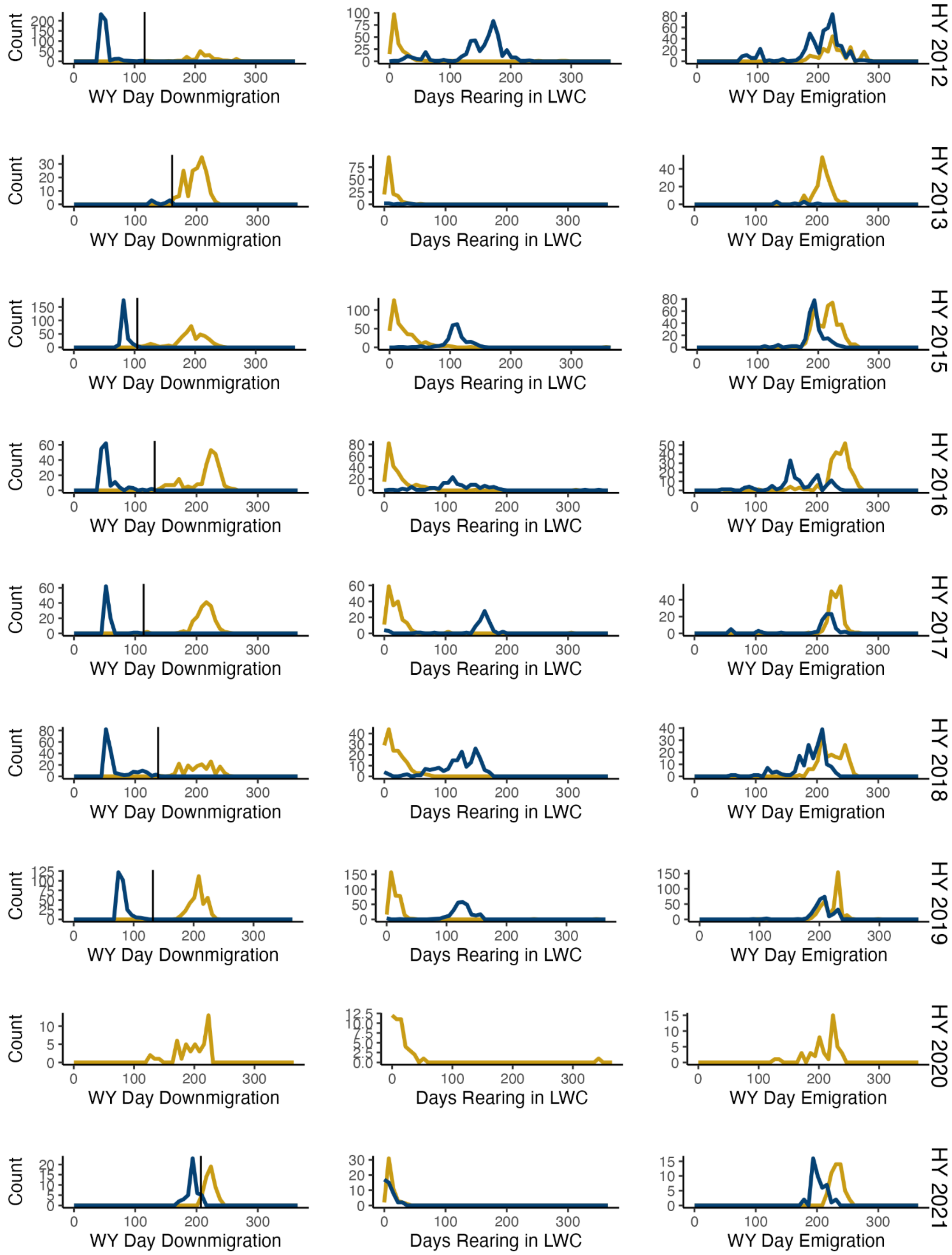


Figure S2: The distribution of timing of down-migration (water year day; left), residence time in lower Willow Creek (center), and timing of emigration from Willow Creek to the Russian River estuary (water year day; right) for each cohort (hatch year) of non-natal (blue) and natal (yellow) rearing fish. Vertical lines in the left column show the thresholds for classifying non-natal vs. natal rearing based on finite Gaussian mixture models.

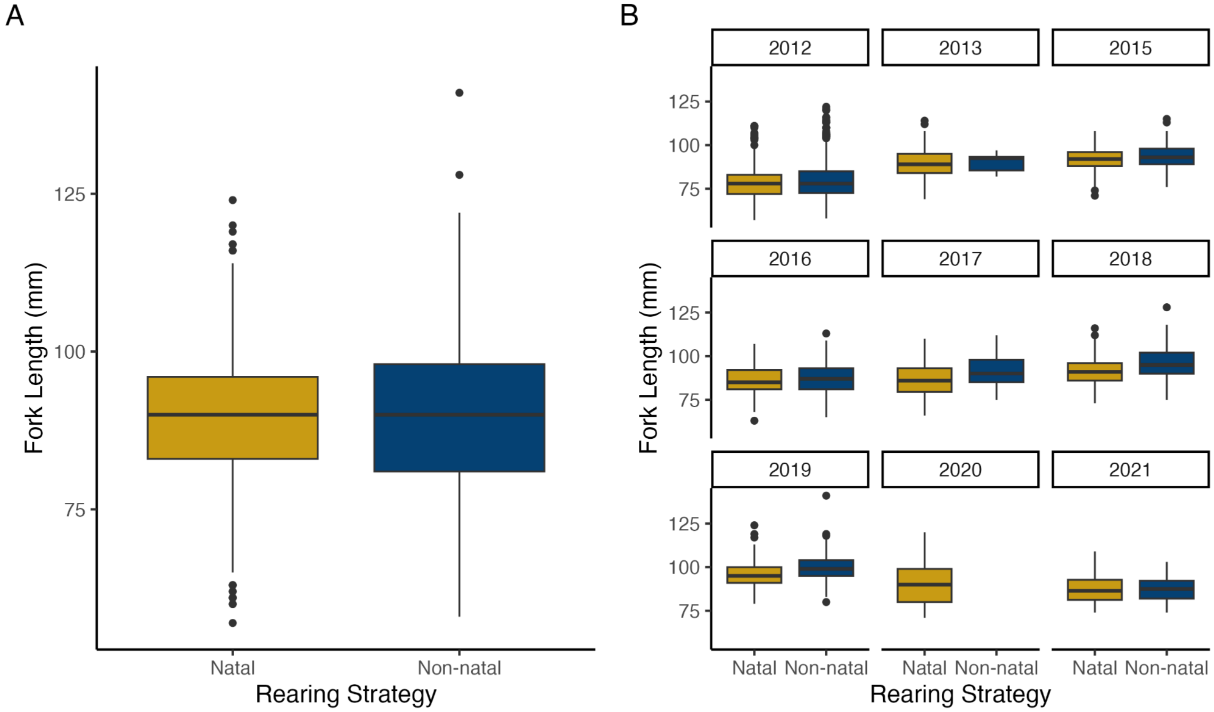


Figure S3: Fork length (mm) of juvenile coho salmon measured at the hatchery prior to release for each rearing strategy (A) aggregated across cohorts and (B) separately by cohort (hatch year).

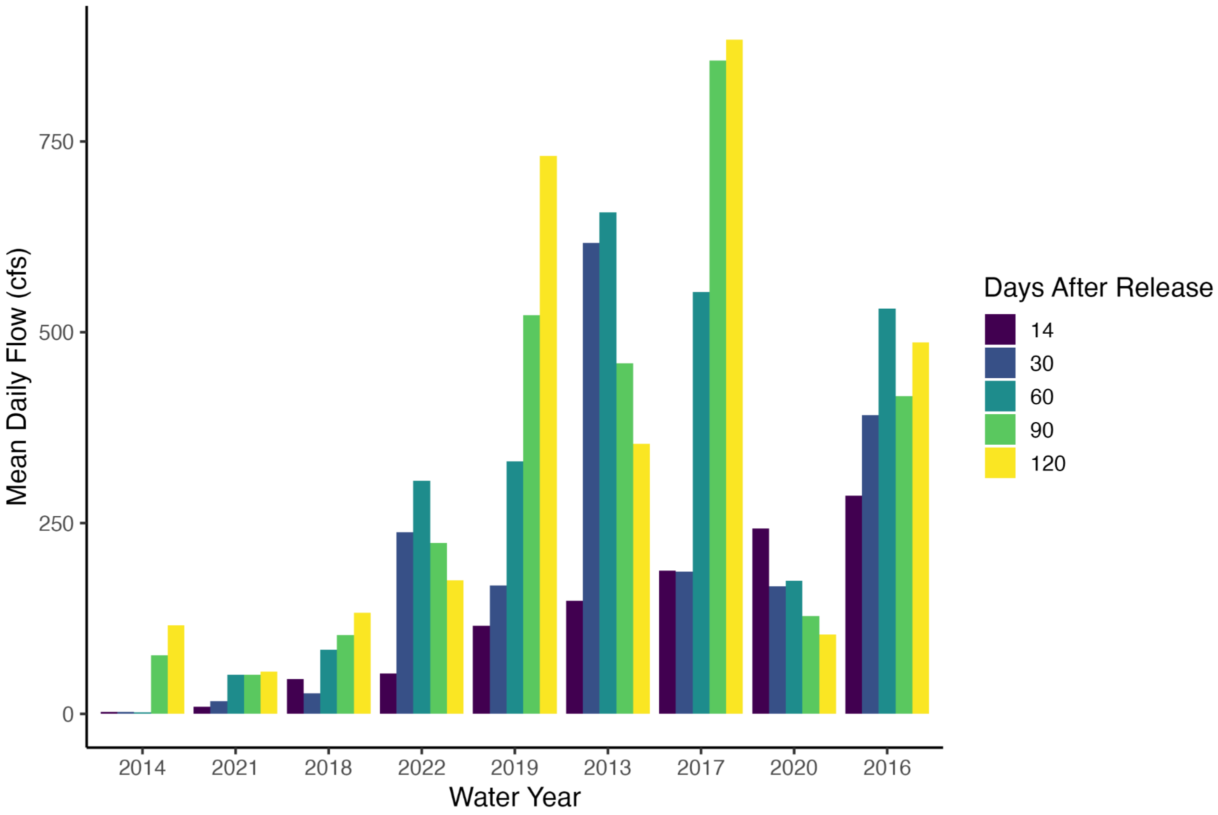


Figure S4: Comparison of 14-, 30-, 60-, 90-, and 120-day flow windows following release (arranged in increasing order of mean daily flow for the 14-day window), showing that years with low flow immediately following release also have low across broader time windows.

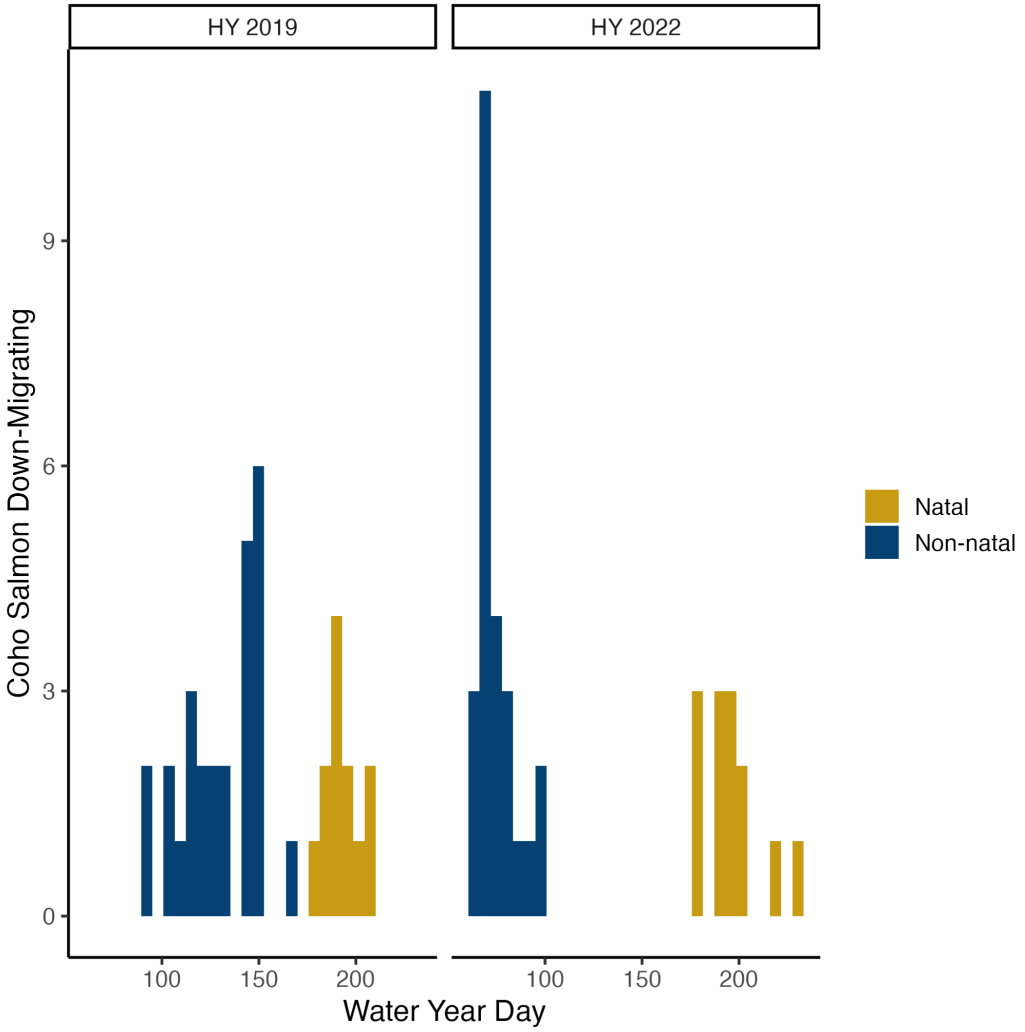


Figure S5. Histogram of down-migration timing of wild juvenile Coho Salmon in Willow Creek, including 38 captured in fall 2019 and 38 captured in fall 2022, showing similar bimodal patterns of down-migration as hatchery-origin fish described in the main body.

*Calculating juvenile Coho Salmon abundance for competition analysis*

We used the abundance of juvenile Coho Salmon in the natal habitat as a proxy for the strength of intraspecific competition to test the hypothesis that higher competition drives a greater proportion of non-natal rearing. Estimating juvenile abundance requires estimating the abundance of wild fish in the natal habitat in addition to the known number of hatchery-released fish. To do this, we used data from the downstream migrant trap (DSMT), which recorded wild versus hatchery origin based on coded wire tag detections, to determine the ratio of wild to hatchery fish in the natal habitat for each year. We then applied that ratio to the known number of hatchery fish released in a given year to calculate the number of wild fish, then added the wild fish to hatchery fish to calculate total estimated abundance.
